# Clustering of hypertension and clustering of diabetes at the household level and variations in disease awareness within households in India: findings from a nationally representative household survey

**DOI:** 10.1136/bmjgh-2024-018809

**Published:** 2026-02-16

**Authors:** Sarang Pedgaonkar, Shubham Kumar, Wahengbam Bigyananda Meitei, Aditi Chaudhary, Abhishek Singh

**Affiliations:** 1 Department of Family and Generations, International Institute for Population Sciences, Mumbai, Maharashtra, India; 2 International Institute for Population Sciences, Mumbai, Maharashtra, India; 3 GENDER Project, International Institute for Population Sciences, Mumbai, Maharashtra, India; 4 Department of Public Health and Mortality Studies, International Institute for Population Sciences, Mumbai, Maharashtra, India

**Keywords:** India, Global Health, Public Health, Diabetes, Hypertension

## Abstract

**Objective:**

Despite rising prevalence, very limited evidence is available on the clustering of hypertension and clustering of diabetes at household level in India. This study examines the clustering of hypertension and clustering of diabetes at household level among members aged 15 years and above in India.

**Methods:**

Clustering of hypertension is defined as two or more members of the household having hypertension. Clustering of diabetes is defined as two or more members of the household having diabetes. Clustering was examined in 636 699 households interviewed in the fifth round of the National Family Health Survey 2019–2021. The relationship dyads of clustering and awareness of the diseases within households were also examined.

**Results:**

Two or more members suffered from hypertension in 14.9% households, which contributed to 49.8% of total hypertension cases in India. Diabetes was clustered in 7.7% of households which contributed to 39.3% of total diabetes cases in India. Among households with two diagnosed members, the most common relationship dyad was spouses (53.6% for hypertension and 53.8% diabetes), followed by parent–child (29.8% for hypertension and 28.8% for diabetes). In households with three diagnosed members, the most common dyad was parent–child (44.3% for hypertension and 42.5% for diabetes). Among households with clustering, all the members with disease were unaware in 42.5% of the households for hypertension and 55.5% for diabetes, and *mixed awareness* was seen in 37.9% and 31.4% households for hypertension and diabetes, respectively.

**Conclusion:**

Given the disproportionate amount of India’s total case burden of hypertension and diabetes concentrated within clustered households, our findings underscore the importance of targeting households for interventions of hypertension and diabetes management in addition to interventions targeting individuals. Our findings may equip health systems with information on patterns of concentrated pockets of undiagnosed disease burden within households and may help in designing intensified interventions for rapid progress towards Sustainable Development Goal V.3.4.

**WHAT IS ALREADY KNOWN ON THIS TOPIC**

- Limited evidence exists on familial clustering of hypertension and familial clustering of diabetes in India, with most studies being hospital-based, small-scale and focused on selected population subgroups.
- Few studies in India draw on population-based samples; however, their scope has largely been confined to examining concordance of hypertension among married couples rather than entire households.

**WHAT THIS STUDY ADDS**

- Clustering at household level is evident for both conditions with disproportionate concentration of disease cases in clustered households—two or more members had hypertension in 14.9% of households which accounted for 49.8% of total hypertension cases in India; while for diabetes, 7.7% of households accounted for 39.3% of total diabetes cases in India.
- For both these diseases, the most common relationship dyad among affected family members in households with two diagnosed members was spouses, followed by parent–child, while that in households with three diagnosed members was parent–child, followed by spouses.
- Disease awareness varied even within the members of clustered households: for hypertension in 38% of clustered households, at least one member with hypertension was not aware of own disease status when at least one member from the same household was aware of disease status (*mixed awareness*) and in 43% households, none was aware of own disease status (*all unaware*) and for diabetes, corresponding proportions were 31% and 55%, respectively.

**HOW THIS STUDY MIGHT AFFECT RESEARCH, PRACTICE OR POLICY**

- By providing evidence for clustered distribution of hypertension and diabetes and disproportionate concentration of case burden within clustered households in India, our findings support interventions targeting households and emphasise the urgent necessity for frequent screening and health education among the direct relatives of individuals diagnosed with hypertension or diabetes.
- Insights into the relationship dyads among affected members deepen understanding of epidemiology of hypertension and diabetes in India.
- The study’s research approach can be replicated in other low- and middle-income countries to enhance understanding of non-communicable diseases.

## Introduction

 The ever-increasing prevalence of non-communicable diseases (NCDs) like hypertension and diabetes poses a daunting public health problem in India, with approximately 220 million people suffering from hypertension and 77 million people suffering from diabetes. Around two-thirds of hypertension cases are concentrated in low- and middle-income countries (LMICs).^1^,^3^ India contributes approximately one-sixth to the total cases of hypertension and one-seventh to the total cases of diabetes across the world.^4^,^6^ These diseases are unequally distributed across the states, urban–rural residence and socioeconomic groups in India.^7^,^10^ Moreover, substantial disparities exist in diagnosis, treatment and control of NCDs in India.^11^,^14^ NCDs bring on huge costs to households due to expensive and long-term treatment and can push the households towards poverty.^15^ A socioeconomically deprived and vulnerable population is more harshly affected and bears a disproportionate burden of mortality and morbidity due to the NCDs, impeding progress towards sustainable growth.^2 16^

Occurrence of NCDs, including hypertension and diabetes, depends on complex interaction of genetic, environmental, behavioural and lifestyle factors, known as risk factors. Major modifiable risk factors for NCDs are tobacco use, physical inactivity, unhealthy diet and harmful consumption of alcohol. Several environmental factors, particularly air pollution, contribute to the development of NCDs.^2^ Familial clustering, which refers to the concentration of certain phenomena within families, is frequently used in mortality research.^17 18^ In this study, we are using the term clustering in the context of NCDs, referring to concentration of NCDs within certain families. Familial clustering is typically observed in diseases which have genetic as well as environmental determination. Environmental factors cause familial clustering as family members often experience common environmental exposures and food habits.^19 20^ Even the high-risk behaviours associated with modifiable risk factors for NCDs are influenced by sociocultural and economic factors. These behaviours can be transmitted through human interactions and therefore are likely to be concentrated within families.^21^,^25^ In addition, social transmission of behaviours for modifiable risk factors across generations can lead to intergenerational spread of NCDs.^23^ Familial clustering is therefore expected for hypertension and diabetes, which is substantiated by available literature, though evidence from India and other LMICs is limited.^26^,^41^

A substantial proportion of cases of NCDs, including hypertension and diabetes, are undiagnosed in LMICs in general and India in particular.^11 12^ So, examining familial clustering has several important policy implications. First, identifying household-level clustering can improve our understanding of how these diseases are distributed across a population and highlight households with a disproportionately high disease burden. This approach allows us to assess disease awareness within households. Differences in awareness among household members, together with the presence of multiple affected individuals, may indicate the potential for family-based ‘snowballing’ to facilitate rapid case identification and treatment. Second, apart from shared risks and social transmission of high-risk behaviours and chronic conditions, families often provide immediate support to their members. These same social ties can be harnessed to encourage healthy behaviour changes, foster support and better management of the diseases in terms of seeking and adherence to treatment.^42^,^44^ Therefore, the public health interventions can be more effectively targeted at families in addition to interventions targeting individuals. Third, the presence of familial clustering may indicate multiple members simultaneously suffer from the disease and subsequent complications, increasing the economic burden and need for healthcare and other support services. Fourth, with representative sampling design, studies examining clustering can throw light on distribution of disease across a population; certain sections of population may bear a disproportionately high disease burden. The understanding and identification of familial clustering can help in identifying the concentrated disease burden within a population for rapid outreach of healthcare and proper management and care.

Given these, it is essential to examine the familial clustering of hypertension and diabetes in a large and diverse country like India. Such examination may help in understanding disease awareness in the context of household level clustering and targeting the clustered households for early identification of undiagnosed disease load and its management. Despite the importance of familial clustering of hypertension and diabetes, the evidence on familial clustering of hypertension and diabetes is limited.^31 33 35 37 45^ Though familial aggregation is observed in these studies, these studies are either hospital based or small-scale studies and based on selected population subgroups, which often lack generalisability and policy input. Few studies have examined familial clustering of chronic conditions and hypertension using representative datasets, with most focusing only on concordance of hypertension within married couples.^3239^,^41^ A key study in this area by Patel *et al* was conducted in only four districts of India and focused mainly on concordance of selected chronic conditions among co-residing adults.^32^

Given the limited evidence on household-level clustering of NCDs in LMICs, particularly in India, our study investigates the clustering of hypertension and clustering of diabetes at the household level in India. Our study contributes to the existing literature in several important ways. First, our study examines household level clustering of hypertension and clustering of diabetes using a large-scale nationally representative population-based household survey conducted in 2019–2021. Our study also quantifies the case burden within households exhibiting clustering and maps relationship dyads among affected members to show how hypertension and diabetes are distributed across kinship structures. In addition, our study assessed intrahousehold variation in disease awareness, highlighting opportunities for family-based identification and management. Understanding the distribution of hypertension and diabetes with the unique context of household level clustering will help in curating prevention, detection and control strategies in an effective manner.

## Methods

### Patient and public partnership

Patients were not involved in this research. Patients were not consulted during the study design, the development of outcome measures or the interpretation of results. Patients neither contributed to the writing or editing of this document to ensure readability or accuracy nor to the dissemination of results.

### Data source

Data from the fifth round of the National Family Health Survey (NFHS-5) was used for the study. NFHS-5, akin to the Demographic and Health Survey (DHS), is a nationally representative household survey conducted across all 28 states and 8 union territories of India during June 2019 to April 2021. NFHS-5 adopted a two-stage stratified sampling design in both urban and rural areas. The detailed information on sampling design can be found elsewhere.^10^ A total of 636 699 households were interviewed in NFHS-5, for a response rate of 98%.

In NFHS-5, all members of the surveyed households aged 15 years and above were eligible for blood pressure (BP) and random blood glucose (RBG) measurements. In households with only one eligible member or where only one member consented, measurements were conducted for that member only. These cases were relatively few, accounting for just 4.5% of hypertension measurements and 4.3% of diabetes measurements.

### Analytical sample

Of all the households interviewed, 16 households headed by transgender respondents were excluded from the analysis. A total of 619 832 households were considered for analysis of clustering of hypertension where BP measurement was completed for at least one member of the household and a total of 615 118 households were considered for analysis of clustering of diabetes where RBG measurement was completed for at least one member of the household. We followed the STROBE (Strengthening the Reporting of Observational Studies in Epidemiology) guideline for reporting the study (online supplemental checklist S1).

### Measurement

All biomarkers in NFHS-5 were collected by paramedical personnel who were specifically trained in biomarker collection for 2 weeks followed by field practice of at least 3 days. BP and RBG were measured after completion of the survey questionnaire and obtaining informed consent (online supplemental text S1). The detailed description of the protocols for measurement of BP and RBG in NFHS-5 can be found elsewhere.^46^ All the respondents aged 15 years and above were also asked if she or he was told on two or more occasions by a doctor, nurse or auxiliary nurse midwife that she or he has hypertension or high BP and high blood glucose (online supplemental text S2).^47^

A respondent having systolic BP ≥140 mm Hg or diastolic BP ≥90 mm Hg or taking any medication to lower her/his BP at the time of survey was identified as hypertensive.^48^ A respondent having an RBG level greater than 140 mg/dL or taking any medicines for diabetes was identified as having diabetes (online supplemental text).^10 49^ NFHS-5 data does not allow distinction between type 1 and type 2 diabetes; therefore, we did not differentiate between them in this study.

### Analytical procedure

Household level clustering occurs when a household has two or more members diagnosed with the disease. For example, household level clustering for hypertension occurs when two or more members of a household are diagnosed with hypertension. Likewise, household level clustering for diabetes occurs when two or more members of a household are diagnosed with diabetes. We assessed clustering for hypertension and clustering of diabetes separately (online supplemental text S4).

Further, among the households having two members diagnosed with either hypertension or diabetes at the time of survey, we traced the relationship between the two members by establishing the relationship dyad. Based on the information of the relationship of the household members to the head of the household provided in NFHS-5, we created the relationship dyads between the two household members, namely, spouses, parent–child, grandparent–grandchild, siblings, avuncular, exposed non-genetic overlap relation (ENGOR) and not related. ENGOR includes those relationships among household members which as such do not have genetic overlap but share possible environmental and other household exposures and lifestyle, such as brother-in-law, sister-in-law, parents-in-law. The classification of various relationships of household members with respect to the head of the household into the relation dyads is shown in supplementary material (online supplemental table S1). We applied the same dyad-mapping approach to households with three members diagnosed with either hypertension or diabetes at the time of the survey. In such households, three pairwise dyads were possible, and all were mapped. However, because households with more than three members diagnosed with either hypertension or diabetes at the time of the survey were very few, and the analytical complexity increases substantially with additional members, we did not construct dyads for households with more than three individuals identified with either condition.

During the survey, each of the eligible members in the household was asked whether he or she had been diagnosed with either hypertension or diabetes before the survey. Based on the total number of household members diagnosed with hypertension prior to the survey and the total number of household members identified as having hypertension in the survey, we grouped the households with clustering for hypertension into four categories, namely, *all aware* households (when all of the household members who were identified as having hypertension during the survey also reported being diagnosed with hypertension prior to the survey), *mixed awareness* households (when out of all the household members who were identified as having hypertension during the survey, at least one member has also reported being diagnosed with hypertension prior to the survey and at least one member has reported not being diagnosed with hypertension prior to the survey), *all unaware* households (all of members of household who were identified as having hypertension during the survey also reported not being diagnosed with hypertension prior to the survey). One additional category, *inconsistent,* is also identified where the total number of household members reporting being diagnosed with hypertension prior to the survey is greater than the total number of members identified with hypertension at the time of survey by actual measurement. Inconsistent groups may be due to recall bias or misreporting and hence were not considered for further analysis. Similarly, we grouped the households with clustering for diabetes into four categories*—all aware*, *mixed awareness*, *all unaware* and *inconsistent*. Of these groups, our interest lies more in *all unaware* and *mixed awareness* groups, as these are important for the policy and health system targeting.

We assessed the variation of household level clustering of hypertension and clustering of diabetes by selected characteristics of the head of the household (sex, schooling, caste and religion), number of members aged 15–30 years in the household, 31–50 years in the household, 51–60 years in the household, 61–70 years in the household and 71 years and above in the household, share of 15+ years women in 15+ years members of the household, place of residence and household’s socioeconomic status (wealth quintiles). Schooling of the head of the household was classified into—no schooling, primary, secondary and higher. Caste was classified into—scheduled castes (SCs), scheduled tribes (ST), other backward classes (OBC) and others. The wealth index was estimated through principal component analysis by employing a set of variables representing durable asset ownership, access to utilities and infrastructure and housing characteristics of households surveyed in NFHS-5. In the absence of income or expenditure data, the wealth index is considered a good proxy for economic status of the households in NFHS.^10 50^ We used multinomial logistic regression to assess the association between disease awareness status of members within clustered households and selected household characteristics. As our interest was focused only on awareness, multinomial logistic regression was fitted considering *all aware* as the base outcome.

In all our analyses, we incorporated sampling weights and accounted for complex survey design of NFHS-5 to estimate the CIs. The analysis was done using Stata V.16.^51^

## Results

Online supplemental figure S1 shows the selection of analytical sample. BP of any member could not be measured in 16 851 households because participants did not give consent, were not available for measurement or had open sore or wound or irritations on both hands that prevented fitting of cuff of BP monitor. Similarly, RBG of any member could not be measured in 21 565 households because participants did not give consent or were not available for measurement. Online supplemental table S2 shows the characteristics of the sampled households. The majority of the households were residing in rural areas (66.8%) and about one-third (33.2%) were residing in urban areas. The majority of households were headed by men (82.6%). About half of the household heads have completed secondary schooling or above, whereas around 28.5% heads were without schooling or less than 5 years of schooling. Caste-wise distribution showed that 41.6% of the household heads were OBC, 21.7% SC, 9.5% ST and around one-fourth belonged to other castes. Among the households in which BP or RBG could not be measured for any member, the majority resided in urban areas (60.5% for hypertension and 56.9% for diabetes) and belonged to the richest wealth quintile (44.7% for hypertension and 40.6% for diabetes) (online supplemental table S3).

Overall, in 14.9% of the households, two or more members aged 15 years or above were identified with hypertension in NFHS-5. Similarly, in 7.7% of households, two or more members aged 15 years or above were identified with diabetes. The clustered households were burdened with about 49.8% of the total cases of hypertension and 39.3% of the total cases of diabetes in India. In 51.4% of households, not a single member was identified with hypertension and among 66.5% of households, not a single member was identified with diabetes, whereas only one member was identified with hypertension in 33.7% of households and with diabetes in 25.8% of households (table 1). In other words, roughly half of all hypertension cases in India occurred in households with at least one other hypertensive member, while the other half were the only hypertensive individual in their household. Similarly, about 40% of diabetes cases were in households with another diabetic member, whereas the remaining 60% were the sole diabetic in their household.

**Table 1:** Percentage distribution of households with hypertensive and diabetic members and proportion of total hypertension and diabetes burden in India

Number of HH members with disease	Hypertension				Diabetes			
	%	95% CI	N	% of the total burden of cases	%	95% CI	N	% of the total burden of cases
0	51.4	(51.3 to 51.5)	318 680		66.5	(66.4 to 66.6)	409 148	
1	33.7	(33.6 to 33.8)	208 688	50.2	25.8	(25.7 to 25.9)	158 800	60.7
2+	14.9	(14.8 to 15.0)	92 464	49.8	7.7	(7.6 to 7.7)	47 170	39.3
Total			619 832				615 118	

HH, household.

Table 2 shows the clustering of hypertension and clustering of diabetes, respectively, by selected characteristics of the household. Higher clustering for both the diseases was observed among households residing in urban areas (17.1% for hypertension and 9.5% for diabetes) than rural areas (13.9% for hypertension and 6.8% for diabetes). The clustering for both hypertension and diabetes was highest among households from the wealthiest quintile (22.2% and 12.2%, respectively) and declined progressively to the lowest level in the poorest wealth quintile (8.8% and 4.2%, respectively). A similar gradient was observed by education, with highest clustering among households with heads having schooling higher than secondary level (17% for hypertension and 9.6% for diabetes) and lowest among those with no schooling or less than 5 years of schooling (12.8% for hypertension and 5.9% for diabetes). Clustering was higher in households headed by men (16.2% for hypertension and 8.4% for diabetes) than that by women (9.1% for hypertension and 4.3% for diabetes). Clustering was highest among households headed by individuals from other castes (17.1% for hypertension and 9.6% for diabetes), followed by OBC (14.9% for hypertension and 7.8% for diabetes) and lowest among ST (12.9% for hypertension and 4.9% for diabetes).

**Table 2:** Per cent of households with the number of hypertensive and diabetic cases in the household by selected household characteristics

	Hypertension							Diabetes						
	HH with no cases	95% CI	HH with 1 case	95% CI	HH with 2+ cases	95% CI	N	HHs with no cases	95% CI	HHs with 1 case	95% CI	HHs with 2+ cases	95% CI	N
Place of residence														
Urban	47.8	(47.6 to 48.1)	35.1	(34.8 to 35.3)	17.1	(16.9 to 17.3)	199 117	61.9	(61.7 to 62.2)	28.6	(28.3 to 28.8)	9.5	(9.4 to 9.7)	197 137
Rural	53.1	(53.0 to 53.3)	33.0	(32.9 to 33.1)	13.9	(13.8 to 14.0)	420 715	68.7	(68.6 to 68.8)	24.5	(24.4 to 24.6)	6.8	(6.7 to 6.9)	417 981
Sex of the HH head														
Male	50.5	(50.4 to 50.7)	33.3	(33.2 to 33.5)	16.2	(16.0 to 16.2)	511 439	65.4	(65.3 to 65.6)	26.2	(26.1 to 26.3)	8.4	(8.3 to 8.4)	508 488
Female	55.6	(55.3 to 55.9)	35.3	(35.0 to 35.6)	9.1	(8.9 to 9.3)	108 393	71.7	(71.4 to 72.0)	24.0	(23.7 to 24.2)	4.3	(4.2 to 4.4)	106 630
Schooling of HH head														
No schooling	52.9	(52.6 to 53.1)	34.3	(34.1 to 34.5)	12.8	(12.7 to 13.0)	177 760	70.0	(69.8 to 70.2)	24.1	(23.9 to 24.3)	5.9	(5.8 to 6.0)	175 991
Primary	50.7	(50.4 to 50.9)	34.0	(33.8 to 34.3)	15.3	(15.1 to 15.5)	116 633	65.8	(65.5 to 66.1)	26.4	(26.2 to 26.7)	7.8	(7.6 to 7.9)	115 889
Secondary	51.1	(50.9 to 51.3)	33.2	(33.0 to 33.4)	15.7	(15.5 to 15.8)	260 264	65.4	(65.2 to 65.6)	26.2	(26.1 to 26.4)	8.3	(8.2 to 8.4)	258 660
Higher	50.0	(49.6 to 50.4)	33.0	(32.6 to 33.4)	17.0	(16.7 to 17.3)	65 175	62.7	(62.3 to 63.1)	27.6	(27.3 to 28.0)	9.6	(9.4 to 9.9)	64 578
Caste of HH head														
Scheduled tribe	54.6	(54.3 to 54.9)	32.5	(32.2 to 32.8)	12.9	(12.7 to 13.1)	59 878	74.4	(74.1 to 74.6)	20.8	(20.5 to 21.0)	4.9	(4.8 to 5.0)	59 571
Scheduled caste	54.2	(53.9 to 54.5)	32.6	(32.4 to 32.9)	13.2	(13.0 to 13.4)	135 385	69.4	(69.2 to 69.7)	24.3	(24.0 to 24.5)	6.3	(6.1 to 6.4)	134 483
Other backward classes	51.4	(51.2 to 51.6)	33.7	(33.5 to 33.9)	14.9	(14.7 to 15.0)	258 217	66.0	(65.8 to 66.2)	26.2	(26.0 to 26.3)	7.8	(7.7 to 7.9)	256 167
Others	48.0	(47.7 to 48.2)	34.9	(34.6 to 35.1)	17.1	(16.9 to 17.3)	166 352	62.1	(61.8 to 62.3)	28.4	(28.1 to 28.6)	9.6	(9.4 to 9.7)	164 897
Religion of HH head														
Other religion	49.9	(49.1 to 50.6)	34.9	(34.2 to 35.6)	15.2	(14.7 to 15.7)	8160	71.0	(70.4 to 71.7)	23.3	(22.7 to 24.0)	5.6	(5.3 to 6.0)	8106
Hindu	51.6	(51.5 to 51.8)	33.6	(33.5 to 33.7)	14.8	(14.7 to 14.9)	509 748	66.8	(66.6 to 66.9)	25.7	(25.5 to 25.8)	7.6	(7.5 to 7.7)	506 361
Muslim	53.7	(53.3 to 54.1)	32.7	(32.3 to 33.0)	13.6	(13.4 to 13.9)	75 470	66.2	(65.8 to 66.5)	25.9	(25.6 to 26.2)	7.9	(7.7 to 8.1)	74 503
Christian	45.5	(45.1 to 46.0)	37.2	(36.8 to 37.6)	17.3	(17.0 to 17.6)	17 445	59.0	(58.6 to 59.5)	30.9	(30.4 to 31.3)	10.1	(9.9 to 10.4)	17 250
Sikh	32.7	(31.9 to 33.5)	37.9	(37.1 to 38.8)	29.4	(28.6 to 30.1)	9009	64.8	(63.9 to 65.6)	27.3	(26.5 to 28.0)	8.0	(7.5 to 8.4)	8899
Wealth quintile														
Poorest	60.8	(60.6 to 61.1)	30.4	(30.1 to 30.6)	8.8	(8.6 to 8.9)	130 919	74.9	(74.7 to 75.1)	21.0	(20.8 to 21.2)	4.2	(4.1 to 4.3)	129 739
Poorer	56.1	(55.8 to 56.3)	32.2	(32.0 to 32.5)	11.7	(11.5 to 11.9)	126 015	70.9	(70.6 to 71.1)	23.6	(23.4 to 23.8)	5.5	(5.4 to 5.7)	125 177
Middle	51.1	(50.8 to 51.4)	34.2	(34.0 to 34.5)	14.7	(14.5 to 14.9)	125 443	66.6	(66.4 to 66.9)	26.0	(25.7 to 26.2)	7.4	(7.2 to 7.5)	124 663
Richer	46.0	(45.7 to 46.3)	35.9	(35.6 to 36.1)	18.1	(17.9 to 18.3)	121 713	61.9	(61.6 to 62.1)	28.5	(28.2 to 28.7)	9.7	(9.5 to 9.9)	120 865
Richest	41.7	(41.4 to 42.0)	36.0	(35.7 to 36.3)	22.2	(22.0 to 22.5)	115 742	57.1	(56.8 to 57.4)	30.8	(30.5 to 31.1)	12.2	(12.0 to 12.4)	114 675
Total	51.4	(51.3 to 51.5)	33.7	(33.6 to 33.8)	14.9	(14.8 to 15.0)	619 832	66.5	(66.4 to 66.6)	25.8	(25.7 to 25.9)	7.7	(7.6 to 7.7)	615 118

HH, household.

**Figure 1:**
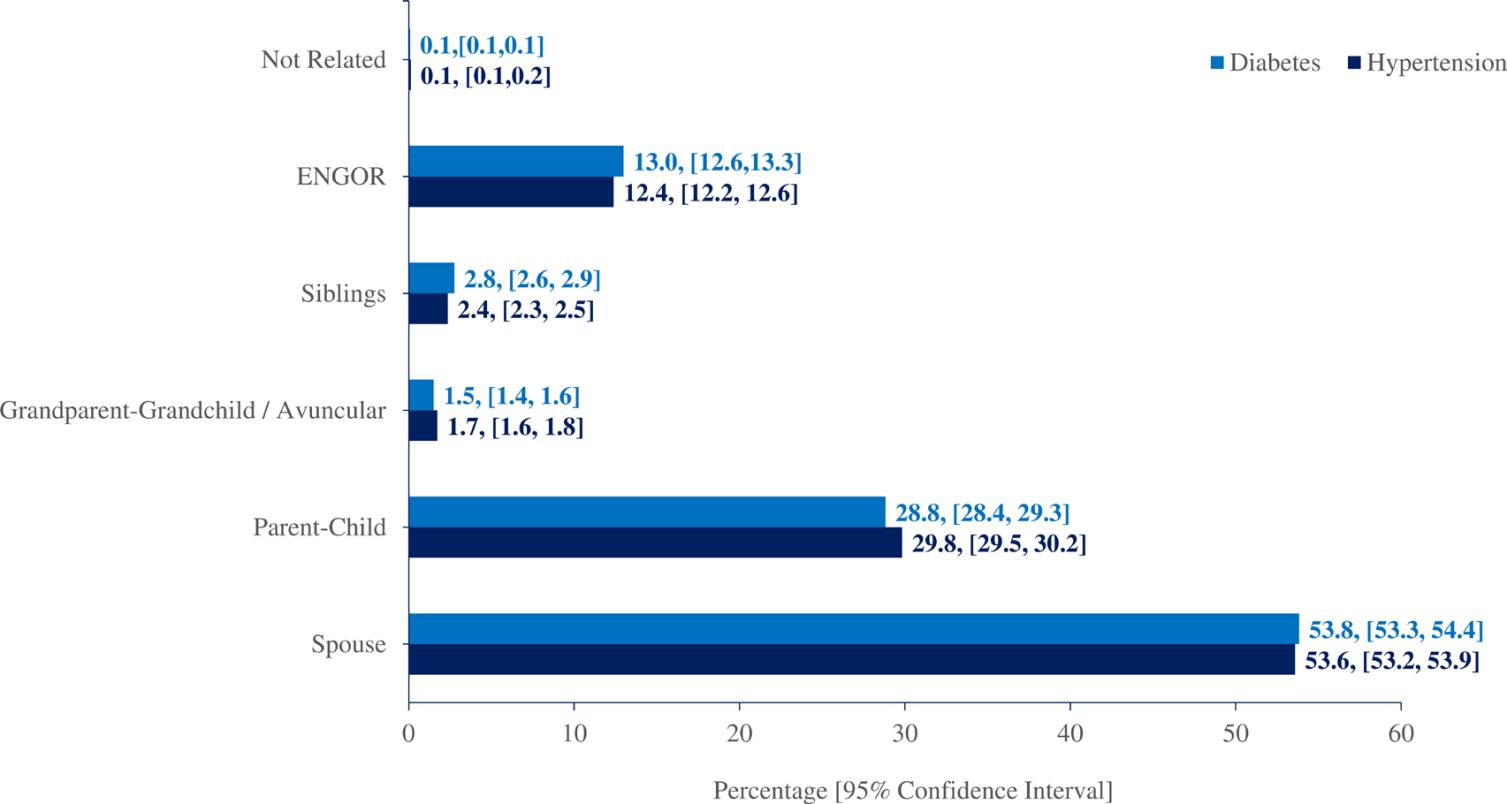
Percentage distribution of relationship dyads in households having two members diagnosed with hypertension and diagnosed with diabetes. ENGOR, exposed non-genetic overlap relation.

Figure 1 shows the relationship dyads among the affected household members where two members were diagnosed with either hypertension or diabetes. Spouses were the most common dyad observed for both hypertension (53.6%) and diabetes (53.8%). This was followed by parent–child (29.8% for hypertension and 28.8% for diabetes), ENGOR (12.4% for hypertension and 13% for diabetes), siblings (2.4% for hypertension and 2.8% for diabetes) and grandparent–grandchild/avuncular (1.7% for hypertension and 1.5% for diabetes). The relationship dyads among affected members in households where three members were diagnosed with either hypertension or diabetes are shown in table 3. In these households, the most common dyad was parent–child (44.3% for hypertension and 42.5% for diabetes), followed by spouses (25.9% for hypertension and 26.4% for diabetes) and ENGOR (20.5% for hypertension and 22.1% for diabetes).

**Table 3:** Percentage distribution of relationship dyads in household having three members diagnosed with hypertension and diagnosed with diabetes

	First and second member			First and third member			Second and third member			Total N	%
	N	%	95% CI	N	%	95% CI	N	%	95% CI		
Hypertension											
Spouse	8954	59.5	(58.8 to 60.3)	138	0.9	(0.8 to 1.1)	2577	17.1	(16.5 to 17.7)	11 670	25.9
Parent–child	4292	28.5	(27.8 to 29.3)	9647	64.1	(63.4 to 64.9)	6065	40.3	(39.5 to 41.1)	20 003	44.3
Grandparent–grandchild/avuncular	150	1.0	(0.8 to 1.2)	592	3.9	(3.6 to 4.2)	927	6.2	(5.8 to 6.5)	1669	3.7
Siblings	461	3.1	(2.8 to 3.3)	555	3.7	(3.4 to 4.0)	1434	9.5	(9.1 to 10.0)	2450	5.4
ENGOR	1177	7.8	(7.4 to 8.3)	4070	27.1	(26.4 to 27.8)	3999	26.6	(25.9 to 27.3)	9245	20.5
Not related	6	0.0	(0.0 to 0.1)	37	0.2	(0.2 to 0.3)	38	0.3	(0.2 to 0.3)	80	0.2
Total	15 039			15 039			15 039			45 117	
Diabetes											
Spouse	3033	59.9	(58.5 to 61.2)	59	1.2	(0.9 to 1.5)	917	18.1	(17 to 19.2)	4010	26.4
Parent–child	1420	28.0	(26.8 to 29.3)	3073	60.6	(59.3 to 62)	1964	38.8	(37.4 to 40.1)	6457	42.5
Grandparent–grandchild/avuncular	36	0.7	(0.5 to 0.9)	203	4.0	(3.5 to 4.6)	277	5.5	(4.8 to 6.1)	516	3.4
Siblings	131	2.6	(2.2 to 3)	211	4.2	(3.6 to 4.7)	492	9.7	(8.9 to 10.5)	834	5.5
ENGOR	441	8.7	(7.9 to 9.5)	1511	29.8	(28.6 to 31.1)	1404	27.7	(26.5 to 28.9)	3356	22.1
Not Related	6	0.1	(0 to 0.2)	10	0.2	(0.1 to 0.3)	12	0.2	(0.1 to 0.4)	28	0.2
Total	5067			5067			5067			15 201	

ENGOR, exposed non-genetic overlap relation.

Awareness of hypertension among the members identified with hypertension in clustered households by selected characteristics of households is shown in table 4. Among clustered households, 42.5% of households were *all unaware,* 17.8% were *all aware* and 37.9% were of *mixed awareness*. For diabetes, 55.5% households were *all unaware*, 12.6% households were *all aware* and 31.4% were *mixed awareness*. For both diseases, the proportion of *all unaware households* was higher in households residing in rural areas (46.5% for hypertension and 61.7% for diabetes) than those in urban areas (35.5% for hypertension and 46% for diabetes), households with low schooling of household head (50.8% for hypertension and 66.2% for diabetes) than those with higher level of schooling (32.6% for hypertension and 45.5% for diabetes), poorest households (60.3% for hypertension and 78.5% for diabetes) than richest households (31.6% for hypertension and 41.2% for diabetes) and households with head from ST (62.4% for hypertension and 72.6% for diabetes) than those from SC (46.7% for hypertension and 59.9% for diabetes), OBC (42.3% for hypertension and 53.4% for diabetes) and others (34.7% for hypertension and 52.5% for diabetes). The pattern for *mixed awareness* households and *all aware* households is opposite to that of *all unaware* households. For both diseases, the proportion of *mixed awareness* households was higher in households residing in urban areas, households with heads of the household having higher schooling and richest households.

**Table 4:** Per cent of households with hypertension and diabetes awareness among the households with two or more cases by selected household characteristics

	Inconsistent	95% CI	All aware	95% CI	Mixed awareness	95% CI	All unaware	95% CI	N
Hypertension									
Place of residence									
Urban	1.7	(1.6 to 1.9)	22.3	(21.8 to 22.8)	40.4	(39.8 to 41.0)	35.5	(35.0 to 36.1)	34 565
Rural	2.0	(1.9 to 2.1)	15.2	(14.9 to 15.4)	36.4	(36.0 to 36.7)	46.5	(46.2 to 46.9)	59 305
Sex of the HH head									
Male	1.9	(1.8 to 2.0)	17.8	(17.6 to 18.1)	37.6	(37.3 to 37.9)	42.6	(42.3 to 43.0)	83 847
Female	1.6	(1.3 to 1.8)	17.5	(16.7 to 18.2)	39.9	(38.9 to 40.8)	41.1	(40.1 to 42.1)	10 023
Schooling of HH head									
No schooling	1.7	(1.5 to 1.8)	12.9	(12.4 to 13.3)	34.7	(34.1 to 35.3)	50.8	(50.2 to 51.4)	23 124
Primary	1.5	(1.3 to 1.6)	16.4	(15.8 to 16.9)	37.9	(37.2 to 38.6)	44.3	(43.6 to 45.0)	18 135
Secondary	2.0	(1.8 to 2.1)	19.2	(18.8 to 19.5)	39.2	(38.7 to 39.6)	39.7	(39.2 to 40.2)	41 385
Higher	2.7	(2.4 to 3.0)	25.3	(24.4 to 26.1)	39.4	(38.5 to 40.4)	32.6	(31.7 to 33.5)	11 226
Caste of HH head									
Scheduled tribe	0.9	(0.7 to 1.0)	9.1	(8.7 to 9.5)	27.7	(27.0 to 28.3)	62.4	(61.6 to 63.1)	7830
Scheduled caste	1.7	(1.5 to 1.9)	14.7	(14.2 to 15.2)	36.9	(36.1 to 37.6)	46.7	(45.9 to 47.5)	18 104
Other backward classes	2.0	(1.9 to 2.2)	17.4	(17.0 to 17.8)	38.3	(37.8 to 38.8)	42.3	(41.8 to 42.8)	39 000
Others	2.1	(1.9 to 2.2)	22.6	(22.1 to 23.1)	40.6	(40.0 to 41.2)	34.7	(34.2 to 35.3)	28 936
Religion of HH head									
Other religion	1.0	(0.7 to 1.4)	16.4	(15.1 to 17.7)	41.8	(40.1 to 43.6)	40.8	(39.1 to 42.5)	1258
Hindu	1.8	(1.7 to 1.9)	16.9	(16.7 to 17.2)	37.5	(37.1 to 37.8)	43.8	(43.4 to 44.1)	76 426
Muslim	1.9	(1.6 to 2.2)	20.7	(19.8 to 21.5)	38.1	(37.2 to 39.1)	39.3	(38.3 to 40.3)	10 439
Christian	1.3	(1.1 to 1.6)	28.9	(27.8 to 29.9)	38.6	(37.5 to 39.7)	31.2	(30.2 to 32.3)	3061
Sikh	4.5	(3.8 to 5.1)	19.2	(18.0 to 20.5)	44.3	(42.7 to 45.8)	32.0	(30.6 to 33.5)	2685
Wealth quintile									
Poorest	1.0	(0.8 to 1.2)	11.4	(10.9 to 11.9)	27.3	(26.6 to 28.1)	60.3	(59.5 to 61.1)	11 679
Poorer	1.9	(1.7 to 2.1)	12.4	(11.9 to 12.9)	34.3	(33.6 to 35.0)	51.4	(50.6 to 52.1)	14 968
Middle	1.7	(1.5 to 1.8)	15.4	(14.9 to 15.9)	38.5	(37.8 to 39.2)	44.4	(43.7 to 45.1)	18 714
Richer	1.8	(1.6 to 2.0)	19.3	(18.8 to 19.8)	40.6	(39.9 to 41.2)	38.3	(37.7 to 39.0)	22 371
Richest	2.5	(2.3 to 2.7)	24.2	(23.6 to 24.7)	41.7	(41.1 to 42.4)	31.6	(31.0 to 32.2)	26 137
Total	1.9	(1.8 to 2.0)	17.8	(17.6 to 18.0)	37.9	(37.5 to 38.2)	42.5	(42.2 to 42.8)	93 870
Diabetes									
Place of residence									
Urban	0.5	(0.4 to 0.7)	17.2	(16.5 to 17.8)	36.2	(35.4 to 37.1)	46.0	(45.2 to 46.9)	16 307
Rural	0.6	(0.5 to 0.7)	9.5	(9.1 to 9.8)	28.2	(27.7 to 28.7)	61.7	(61.1 to 62.3)	24 700
Sex of the HH head									
Male	0.6	(0.5 to 0.7)	12.6	(12.3 to 13.0)	31.3	(30.8 to 31.8)	55.5	(54.9 to 56.0)	37 002
Female	0.4	(0.2 to 0.6)	11.9	(10.9 to 12.9)	32.2	(30.8 to 33.6)	55.6	(54.0 to 57.1)	4005
Schooling of HH head									
No schooling	0.4	(0.3 to 0.6)	8.1	(7.5 to 8.6)	25.3	(24.5 to 26.2)	66.2	(65.2 to 67.1)	9024
Primary	0.8	(0.6 to 1.0)	11.2	(10.5 to 11.9)	30.5	(29.5 to 31.5)	57.5	(56.4 to 58.6)	7846
Secondary	0.5	(0.4 to 0.6)	13.5	(13.0 to 14.0)	33.6	(33.0 to 34.3)	52.3	(51.6 to 53.0)	18 739
Higher	0.8	(0.5 to 1.0)	18.7	(17.6 to 19.8)	35.0	(33.7 to 36.4)	45.5	(44.1 to 46.9)	5398
Caste of HH head									
Scheduled tribe	0.3	(0.2 to 0.5)	6.4	(5.7 to 7.0)	20.7	(19.7 to 21.8)	72.6	(71.4 to 73.7)	2528
Scheduled caste	0.6	(0.4 to 0.8)	9.7	(9.0 to 10.4)	29.8	(28.7 to 30.8)	59.9	(58.7 to 61.1)	7349
Other backward classes	0.7	(0.5 to 0.8)	13.6	(13.1 to 14.2)	32.3	(31.6 to 33.0)	53.4	(52.7 to 54.2)	17 422
Others	0.5	(0.4 to 0.7)	13.8	(13.2 to 14.4)	33.1	(32.3 to 33.9)	52.5	(51.7 to 53.4)	13 708
Religion of HH head									
Other religion	0.1	(0.0 to 0.2)	12.0	(9.7 to 14.3)	32.8	(29.5 to 36.0)	55.2	(51.7 to 58.7)	396
Hindu	0.6	(0.5 to 0.7)	11.9	(11.5 to 12.2)	30.9	(30.4 to 31.5)	56.6	(56.0 to 57.1)	33 354
Muslim	0.6	(0.3 to 0.8)	12.7	(11.7 to 13.6)	31.6	(30.3 to 33.0)	55.1	(53.7 to 56.5)	5120
Christian	0.9	(0.6 to 1.2)	26.2	(24.6 to 27.8)	36.8	(35.1 to 38.6)	36.1	(34.3 to 37.8)	1520
Sikh	0.6	(0.2 to 1.1)	14.4	(12.3 to 16.5)	39.3	(36.4 to 42.2)	45.7	(42.7 to 48.6)	617
Wealth quintile									
Poorest	0.4	(0.2 to 0.5)	5.0	(4.4 to 5.6)	16.1	(15.1 to 17.1)	78.5	(77.4 to 79.6)	4684
Poorer	0.5	(0.4 to 0.7)	6.4	(5.8 to 6.9)	22.6	(21.6 to 23.5)	70.5	(69.5 to 71.6)	6029
Middle	0.5	(0.3 to 0.7)	9.6	(9.0 to 10.3)	30.5	(29.5 to 31.5)	59.4	(58.3 to 60.4)	7991
Richer	0.6	(0.4 to 0.8)	13.6	(12.9 to 14.3)	35.9	(34.9 to 36.8)	49.9	(48.9 to 50.9)	10 167
Richest	0.8	(0.6 to 0.9)	19.6	(18.8 to 20.3)	38.5	(37.6 to 39.4)	41.2	(40.2 to 42.1)	12 135
Total	0.6	(0.5 to 0.7)	12.6	(12.2 to 12.9)	31.4	(30.9 to 31.8)	55.5	(55.0 to 55.9)	41 007

HH, household.

Online supplemental table S4 shows the results of disease awareness status of members within clustered households and selected household characteristics using multinomial regression with *all aware* households being the reference category. For both hypertension clustering and diabetes clustering, households were more likely to belong to *all unaware* and *mixed awareness* categories with higher number of members in the age group 15–60 years. The households with more members in the age group 61–70 years were more likely to belong to the *all aware* category; however, the association was statistically significant only in the case of diabetes clustering. Households with a higher share of women members in the age group of 15 years and above were less likely to belong to *all unaware* (Relative Risk Ratio [RRR] 0.99) and *mixed awareness* (RRR 0.99) categories only for hypertension. Rural households were more likely to belong to the *all unaware* category for both hypertension (RRR 1.28) and diabetes (RRR 1.39). The wealthier households were less likely to belong to *all unaware* and *mixed awareness* categories for both hypertension and diabetes. For hypertension, the RRR for richest households was 0.49 for *mixed awareness* and 0.17 for *all unaware,* and that for diabetes was 0.39 and 0.10, respectively. For hypertension, the households headed by individuals from other castes or OBC were less likely to belong to *all unaware* (RRR 0.35 and 0.44, respectively) and *mixed awareness* categories (RRR 0.63 and 0.72, respectively). For diabetes, households with heads from OBC (RRR 0.41) or other castes (RRR 0.58) were less likely to belong to the *all unaware* category. Households with a higher level of schooling of the household head were less likely to belong to the *all unaware* category for both hypertension (RRR 0.71) and diabetes (RRR 0.64), whereas associations with *mixed awareness* were not statistically significant for both diseases. The religion of the household head was not significantly associated with awareness status of the clustered households for both diseases.

## Discussion

Our study is, to our knowledge, the first to document substantial household level clustering of hypertension and clustering of diabetes, using a nationally representative large household survey in India. Although only 14.9% of Indian households had two or more members with hypertension, and 7.7% had two or more members with diabetes, these relatively small groups accounted for a disproportionately large share of cases. Specifically, such households contributed 49.8% of all hypertension cases and 39.3% of all diabetes cases in India. This striking imbalance highlights that a minority of households bear a disproportionate burden of these conditions. These findings are in agreement with the unequal distribution of hypertension and diabetes and their behavioural risk factors as reported in previous studies.^7 8 24^ Our findings also align well with the clustering of behavioural risk factors and multiple risk factors in India and other LMICs.^52^,^55^ While familial aggregation of hypertension and diabetes has been documented in studies from various global contexts,^27^,^3034 56^ our study is likely the first to quantify, at a national scale, the proportion of India’s total hypertension and diabetes cases that are concentrated within clustered households. Coming from the most populous and diverse country also makes our study findings both unique and novel.

The identification of specific relationship dyads affected by hypertension or diabetes, like spouses, parent–child pairs, ENGOR and sibling pairs, highlights the cumulative effects and complex interactions of genetic predispositions, shared environmental exposures, behavioural factors and lifestyle contributing to clustering of diseases within households. Spouses were the most frequently affected dyad, and diseases were also common in ENGOR, which can be attributed to the role of shared environmental exposures, lifestyle and behavioural factors, and other non-modifiable factors like ageing.^26 36 38^ Among clustered households, intergenerational associations were also seen with considerable parent–child pairs being affected. Similar parent–child positive associations for cardiovascular diseases were reported in previous literature.^3456^,^58^ Apart from genetic factors, social transmission of modifiable risk factors may also lead to the spread of diseases across generations.^23^ While we could not include this dimension in our study, future research may explore this dimension in LMICs. However, the identification of relationship dyads within clustered households may help in designing appropriate policy and information materials to impart requisite knowledge.

Our study unveils the drastic inequalities in awareness of disease status among clustered households and provides insight about possible missed opportunities as well as potential targets for health systems to penetrate the expanse of undiagnosed component of these diseases. Our findings add to the already existing literature on geographical and socioeconomic disparities in awareness, treatment and control of NCDs along with unequal healthcare utilisation in India.^11 12 14^ Varied awareness among households highlights the need for targeted interventions at the household level to improve early detection and management of hypertension and diabetes. An important finding from our study is that in around 30%–40% of clustered households, at least one member was unaware of their own diabetic or hypertensive status when at least one member was already aware. This is remarkable and may point towards possible failure of the health system to reach, create requisite awareness, impart relevant knowledge and enrol household members under the coverage of screening and healthcare.

The lack of awareness about disease status is more likely among households with a larger number of members aged 31–60 years, which necessitates interventions for creating awareness and screening at an earlier age. NCDs often manifest at an earlier age among Indians; therefore, interventions targeting younger age groups are more relevant.^59^ With population ageing increasing in India, clustering is likely to get intensified. The chances of *unawareness* were higher among rural and poorer households, households with heads having lower levels of schooling and heads belonging to ST and SC. Thus, the marginalised sections of the population are more likely to get affected more harshly by clustering of hypertension and clustering of diabetes and complications that may arise due to lack of awareness and subsequent lack of treatment. This all poses a greater challenge to healthcare delivery system to reach and provide services for creating awareness, prevention, early diagnosis and adequate management of these conditions. Notably, for hypertension, the likelihood of *all unaware* and *mixed awareness* was slightly lower among households with greater share of women members in the age group 15 years and above. This finding warrants further investigations.

Our findings carry significant public health implications. The clustered distribution of hypertension and diabetes across Indian households offers clear opportunities for rapid case detection and targeted, intensified interventions. Looking for such *all unaware* and *mixed awareness* households can capture a large share of undiagnosed cases. Even snow-balling from diagnosed cases to their household members will unearth substantial undiagnosed hypertension or diabetes cases. Health education and screening of direct relatives of people with NCDs has also been advocated in previous literature.^33 60^ Evidence from our study reinforces the need for frequent screening and health education for direct relatives of known cases of hypertension or diabetes. The phenomenon of clustering of hypertension and clustering of diabetes within households should be explicitly recognised in the national health programmes and strategies to control these diseases, such as the Indian Hypertension Control Initiative.^61^

A key strength of our study is its use of nationally representative NFHS-5 data, enabling estimates that are generalisable to broad Indian population. By examining hypertension and diabetes in the context of households, this study adds to the understanding of disease distribution and clustering at household level in India. A uniqueness of this study is that being based on a sample drawn from the population enables it to provide quantitative estimation of prevalence of clustering and accumulation of total cases of hypertension and diabetes in India in the clustered households. Additionally, existing studies have measured awareness and familial aggregation based on recall of the index cases for other members of their family.^33 60^ Such studies are likely to be affected by considerable recall bias as respondents have to recall the disease status of the relatives. However, in our analysis, the identification of disease status and clustering of hypertension and diabetes is based on actual measurements and awareness is based on recall for self for all the members of the households who were 15 years or older. These make our findings promising and novel. Moreover, the identification of specific relationship dyads affected by hypertension and diabetes within households enhances our understanding of social dynamics influencing disease clustering. Our study uses the Indian equivalent of the DHS, which is conducted in over 90 LMICs. Use of such data makes our study replicable in other LMICs where data on NCDs is particularly limited.

Our study has few limitations that should be considered. First, the disease awareness is based on self-reported diagnosis, which may introduce some bias by under-reporting or misreporting of disease status. Second, the age of onset of diseases is not known for additional analyses. Third, the estimation of diabetes prevalence is based on RBG. While fasting blood glucose or glycated haemoglobin (HbA1c) may provide more reliable estimates of diabetes, we could not use fasting blood glucose or HbA1c in our analysis as only RBG was collected in NFHS-5. Though it was possible to restrict analysis to respondents with 8 hours of fasting, we avoided it as NFHS-5 was not designed for collection of fasting glucose like other DHS surveys, where respondents were prior informed to remain fasted for 8 hours for fasting blood glucose measurement.^62^ Furthermore, segregating fasting individuals would have reduced the sample size and would have particularly affected within household analysis. Fifth, the relationship dyads are based on the relationship of the members with the household head. Sixth, we were unable to estimate the relationship dyads for households with four or more members diagnosed with either hypertension or diabetes. Such households were very rare, accounting for only 0.54% (for hypertension) and 0.18% (for diabetes) of the sampled households. Additionally, identifying relationship dyads within these households proved to be highly challenging. Finally, we were unable to assess across-disease clustering (eg, one household member with hypertension and another with diabetes), as mixed-condition dyads require an analytical framework that accounts for the joint distribution and correlation structure of both conditions. Nevertheless, understanding such patterns remains an important avenue for future research, especially given the growing relevance of multimorbidity within Indian households.

The study findings are promising for formulating policies to address hypertension and diabetes in India and can also be a resource for other LMICs where hypertension and diabetes can be analysed in a similar manner to our approach. By identifying clustering of hypertension and clustering of diabetes within households, our study stresses the importance of targeting the households for interventions to enhance disease awareness, promote healthy lifestyles, early diagnosis and proper management of these diseases. By studying disease clustering from a population-based sample, our study adds to the knowledge of relationship dyads affected by hypertension and diabetes. Insights gained from this study can go a long way in equipping the health system with better understanding of epidemiology of hypertension and diabetes to curate integrated strategies to prevent, screen, control and manage hypertension and diabetes efficiently for advancing towards Sustainable Development Goals V.3.4 by 2030.

## Supplementary material

10.1136/bmjgh-2024-018809 online supplemental file 1

## Data Availability

Data are available in a public, open access repository.
