# Supplementary material for: Clustering of hypertension and clustering of diabetes at the household level and variations in disease awareness within households in India: findings from a nationally representative household survey
Source: BMJ Glob Health. 2026 Feb 16;11(2):e018809. doi: 10.1136/bmjgh-2024-018809 (PMC12911822; doi:10.1136/bmjgh-2024-018809)
Supplement: online supplemental file 1 [file bmjgh-11-2-s001.docx]

**Supplementary Material**

**Text S1**: Measurement

In NFHS-5, all members of the surveyed households aged 15 years and above were eligible for blood pressure (BP) and random blood glucose (RBG) measurements. All biomarkers in NFHS-5 were collected by paramedical personnel who were specifically trained in biomarker collection for two weeks followed by field practice of at-least three days. NFHS-5 measured blood pressure and blood glucose level of eligible men and women age 15 years or above in the surveyed households using standard equipment. Blood Pressure (BP) was measured using an Omron HEM 8712 automatic digital BP monitor manufactured by Omron Healthcare Vietnam Co. Ltd, Vietnam, in a single home visit after completion of the survey questionnaire and obtaining informed consent. Three measurements of blood pressure were taken, preferably on the left arm, in a sitting position, with a five-minute gap between each measurement. The average of the last two measurements was considered as final measurement for further analysis. Random blood glucose was measured using Accu-Chek Performa glucometer manufactured by Roche Diabetes Care, Inc. USA, on capillary whole blood sample drawn after obtaining informed consent from the respondents. All the standard protocols were followed while measuring the blood pressure and random blood glucose.

**Text S2**: Questionnaire

| **Question Number (Biomarker Questionnaire)** | **Question** | | | **Response** | |
| --- | --- | --- | --- | --- | --- |
| 320 & 420 | Were you told on two or more different occasions by a doctor, nurse, or ANM that you had hypertension or high blood pressure? | | | 1.Yes  2. No | |
| 356 & 455 | Were you told on two or more different occasions by a doctor, nurse, or ANM that your blood glucose level was high? | | | 1.Yes  2. No | |
| 321 & 421 | To lower your blood pressure, are you now taking a prescribed medicine? | | | 1.Yes  2. No | |
| 357 & 456 | To lower your blood glucose level, are you now taking a prescribed medicine? | | | 1.Yes  2. No | |
|  |  |  |  | |  |
| **Measurement** | **Times of reading** | **Systolic reading** | **Diastolic reading** | |  |
| **1** | 317 & 417.  **__ __:__ __** am/pm | 318 & 418.  **___** mmHg | 318 & 418.  **___**mmHg | |  |
| **2** | 324 & 424.  __ __:__ __ am/pm | 325 & 425.  ___ mmHg | 325 & 425.  ___mmHg | |  |
| **3** | 328 & 428.  **__ __:__ __** am/pm | 329 & 429.  **___** mmHg | 329 & 429.  **___**mmHg | |  |
| **4** | Average of last 2 readings | 331 & 431  ___mmHg | 331 & 433  ___mmHg | |  |
|  |  | | | | |
| **Measurement** | **Time of Reading Blood Glucose Reading** | | | | |
| **1** | 373 & 472. 374 & 473  __ __:__ __ am/pm __ __ __mg/dL | | | | |
|  |  | | | | |
| The detailed procedure for blood pressure and blood glucose measurement, weight and height measurement in NFHS-5 can be found in the biomarker manual of NFHS-5 available at <https://www.nfhsiips.in/nfhsuser/manual.php> | | | | | |

**Text S3**: Outcome definitions

| **Outcome** |  | **Definition** |
| --- | --- | --- |
| Hypertension | 1 | 1. SBP ≥140 mm Hg OR DBP ≥ 90 mm Hg, or 2. reported currently taking prescribed medication to control BP |
|  | 0 | Otherwise |
| Diabetes | 1 | 1. RBG >140 mg/dL, or 2. reported currently taking prescribed medication to control blood glucose |
|  | 0 | Otherwise |
| Clustering | 1 | Household-level clustering for a disease occurs when a household has two or more members identified with that particular disease |
|  | 0 | When a household has less than two members identified with a particular disease |
| **Note**: SBP = systolic blood pressure, DBP = diastolic blood pressure, RBG = Random Blood Glucose | | |

**Text S4**: Definition of disease clustering prevalence

| **Statistic** | **Definition** | **Explanation** |
| --- | --- | --- |
| Hypertension clustering prevalence | $\frac{N_{HTN}}{N}\times100$ | $N_{HTN}$= weighted number of households in analysis sample with more than 2 members identified with hypertension, clustering (HTN) =1,  N = weighted number of households in analysis sample |
| Diabetes clustering prevalence | $\frac{N_{DIA}}{N}\times100$ | $N_{DIA}$= weighted number of households in analysis sample with more than 2 members identified with diabetes, clustering (DIA) =1,  N = weighted number of households in analysis sample |
| **Note**: HTN = Hypertension, DIA = Diabetes | | |

**Figure S1:** Flowchart of household selection

Households interviewed in the survey

n = 636,699

Households interviewed

n = 636,683

16 Households headed by transgender excluded

Blood Pressure measured households

n = 619,832

Random blood glucose measured households

n = 615,118

**Figure S1**: Flowchart of household selection

**Table S1**: Relationship dyad matrix: relationships within a household as per relationship of member with the head of the household

|  | **Head** | **Wife / husband** | **Son / daughter** | **Son / daughter-in-law** | **Grandchild** | **Parent** | **Parent-in-law** | **Brother / sister** | **Other relative** | **Adopted / foster child** | **Not related** | **Brother or sister-in-law** | **Niece / nephew** | **Domestic servant** | **Don't know** |
| --- | --- | --- | --- | --- | --- | --- | --- | --- | --- | --- | --- | --- | --- | --- | --- |
| **Head** | NA |  |  |  |  |  |  |  |  |  |  |  |  |  |  |
| **Wife or husband** | 1 | 1 |  |  |  |  |  |  |  |  |  |  |  |  |  |
| **Son / daughter** | 2 | 2 | 4 |  |  |  |  |  |  |  |  |  |  |  |  |
| **Son / daughter-in-law** | 5 | 5 | 1 | 5 |  |  |  |  |  |  |  |  |  |  |  |
| **Grandchild** | 3 | 3 | 2 | 2 | 4 |  |  |  |  |  |  |  |  |  |  |
| **Parent** | 2 | 5 | 3 | 5 | 3 | 1 |  |  |  |  |  |  |  |  |  |
| **Parent-in-law** | 5 | 2 | 3 | 5 | 5 | 5 | 1 |  |  |  |  |  |  |  |  |
| **Brother / sister** | 4 | 5 | 3 | 5 | 5 | 2 | 5 | 4 |  |  |  |  |  |  |  |
| **Other relative** | 5 | 5 | 5 | 5 | 5 | 5 | 5 | 5 | 5 |  |  |  |  |  |  |
| **Adopted / foster child** | 5 | 5 | 5 | 5 | 5 | 5 | 5 | 5 | 5 | 5 |  |  |  |  |  |
| **Not related** | 6 | 6 | 6 | 6 | 6 | 6 | 6 | 6 | 6 | 6 | 6 |  |  |  |  |
| **Brother or sister-in-law** | 5 | 4 | 3 | 5 | 5 | 5 | 5 | 1 | 5 | 5 | 5 | 5 |  |  |  |
| **Niece / nephew** | 3 | 3 | 3 | 5 | 5 | 3 | 5 | 2 | 5 | 5 | 5 | 2 | 4 |  |  |
| **Domestic servant** | 6 | 6 | 6 | 6 | 6 | 6 | 6 | 6 | 6 | 6 | 6 | 6 | 6 | 6 |  |
| **Don't know** | 6 | 6 | 6 | 6 | 6 | 6 | 6 | 6 | 6 | 6 | 6 | 6 | 6 | 6 | 6 |
| **Note**: 1 - Spouse, 2 - Parent-child, 3 - Grandparent-grandchild/Avuncular, 4 - Siblings, 5 - ENGOR, 6 - Not related, NA - Not Applicable (a household can have only one head of the household) | | | | | | | | | | | | | | | |

**Supplementary Table S2**: Characteristics of sampled households

|  | **%** | **N** |
| --- | --- | --- |
| *Place of residence* |  |  |
| Urban | 33.2 | 211,263 |
| Rural | 66.8 | 425,420 |
| *Sex of the HH head* |  |  |
| Male | 82.6 | 525,578 |
| Female | 17.5 | 111,105 |
| *Schooling of HH head* |  |  |
| No schooling | 28.5 | 181,124 |
| Primary | 18.6 | 118,429 |
| Secondary | 41.9 | 267,029 |
| Higher | 11.0 | 70,101 |
| *Caste of HH head* |  |  |
| Scheduled Tribe | 9.5 | 60,541 |
| Scheduled Caste | 21.7 | 137,844 |
| Other Backward Classes | 41.6 | 264,880 |
| Others | 27.2 | 173,419 |
| *Religion of HH head* |  |  |
| Other religion | 1.4 | 8,661 |
| Hindu | 81.9 | 521,475 |
| Muslim | 12.4 | 78,927 |
| Christian | 2.8 | 17,880 |
| Sikh | 1.5 | 9,740 |
| *Wealth quintile* |  |  |
| Poorest | 20.8 | 132,298 |
| Poorer | 20.0 | 127,291 |
| Middle | 20.0 | 127,099 |
| Richer | 19.6 | 124,935 |
| Richest | 19.6 | 125,060 |
| **Total** | **100.0** | **636,683** |
| **Note**: HH-Household | | |

**Table S3**: Percentage distribution of households where blood pressure and random blood glucose measurements were taken for one or more members, for only one member, and not taken for any member

|  | **Hypertension** | | | | | | | |  | **Diabetes** | | | | | | | |
| --- | --- | --- | --- | --- | --- | --- | --- | --- | --- | --- | --- | --- | --- | --- | --- | --- | --- |
|  | **HH where Only One**  **Member Measured** | |  | **HH where One or**  **More Members**  **Measured** | |  | **HH where No**  **Member Measured** | |  | **HH where Only One**  **Member Measured** | |  | **HH where One or**  **More Members**  **Measured** | |  | **HH where No**  **Member Measured** | |
|  | **%** | **N** |  | **%** | **N** |  | **%** | **N** |  | **%** | **N** |  | **%** | **N** |  | **%** | **N** |
| *Place of residence* |  |  |  |  |  |  |  |  |  |  |  |  |  |  |  |  |  |
| Urban | 33.6 | 9,458 |  | 32.1 | 1,99,117 |  | 60.5 | 10,191 |  | 33.8 | 9,020 |  | 32.1 | 1,97,137 |  | 56.9 | 12,270 |
| Rural | 66.4 | 18,672 |  | 67.9 | 4,20,715 |  | 39.5 | 6,660 |  | 66.2 | 17,691 |  | 68.0 | 4,17,981 |  | 43.1 | 9,295 |
| *Sex of the HH head* |  |  |  |  |  |  |  |  |  |  |  |  |  |  |  |  |  |
| Male | 36.6 | 10,294 |  | 82.5 | 5,11,439 |  | 83.5 | 14,071 |  | 36.9 | 9,866 |  | 82.7 | 5,08,488 |  | 80.1 | 17,279 |
| Female | 63.4 | 17,836 |  | 17.5 | 1,08,393 |  | 16.5 | 2,780 |  | 63.1 | 16,845 |  | 17.3 | 1,06,630 |  | 19.9 | 4,286 |
| *Schooling of HH head* |  |  |  |  |  |  |  |  |  |  |  |  |  |  |  |  |  |
| No schooling | 48.4 | 13,625 |  | 28.7 | 1,77,760 |  | 22.5 | 3,791 |  | 48.1 | 12,850 |  | 28.6 | 1,75,991 |  | 25.0 | 5,400 |
| Primary | 17.3 | 4,867 |  | 18.8 | 1,16,633 |  | 13.0 | 2,195 |  | 17.2 | 4,602 |  | 18.8 | 1,15,889 |  | 13.6 | 2,932 |
| Secondary | 24.7 | 6,935 |  | 42.0 | 2,60,264 |  | 40.7 | 6,855 |  | 24.9 | 6,657 |  | 42.1 | 2,58,660 |  | 39.6 | 8,549 |
| Higher | 9.6 | 2,703 |  | 10.5 | 65,175 |  | 23.8 | 4,010 |  | 9.7 | 2,602 |  | 10.5 | 64,578 |  | 21.7 | 4,685 |
| *Caste of HH head* |  |  |  |  |  |  |  |  |  |  |  |  |  |  |  |  |  |
| Scheduled Tribe | 9.2 | 2,578 |  | 9.7 | 59,878 |  | 5.6 | 943 |  | 9.2 | 2,453 |  | 9.7 | 59,571 |  | 5.8 | 1,258 |
| Scheduled Caste | 21.8 | 6,130 |  | 21.8 | 1,35,385 |  | 16.7 | 2,813 |  | 21.8 | 5,817 |  | 21.9 | 1,34,483 |  | 17.2 | 3,709 |
| Other Backward Classes | 42.9 | 12,079 |  | 41.7 | 2,58,217 |  | 40.2 | 6,767 |  | 42.9 | 11,461 |  | 41.7 | 2,56,167 |  | 40.7 | 8,781 |
| Others | 26.1 | 7,343 |  | 26.8 | 1,66,352 |  | 37.6 | 6,328 |  | 26.1 | 6,980 |  | 26.8 | 1,64,897 |  | 36.3 | 7,817 |
| *Religion* |  |  |  |  |  |  |  |  |  |  |  |  |  |  |  |  |  |
| Other religion | 1.6 | 446 |  | 1.3 | 8,160 |  | 2.5 | 420 |  | 1.6 | 429 |  | 1.3 | 8,106 |  | 2.3 | 486 |
| Hindu | 85.3 | 23,989 |  | 82.2 | 5,09,748 |  | 73.3 | 12,346 |  | 85.3 | 22,779 |  | 82.3 | 5,06,361 |  | 73.2 | 15,792 |
| Muslim | 8.2 | 2,314 |  | 12.2 | 75,470 |  | 18.1 | 3,049 |  | 8.3 | 2,218 |  | 12.1 | 74,503 |  | 18.4 | 3,958 |
| Christian | 4.2 | 1,171 |  | 2.8 | 17,445 |  | 2.7 | 446 |  | 4.1 | 1,098 |  | 2.8 | 17,250 |  | 2.9 | 623 |
| Sikh | 0.8 | 210 |  | 1.5 | 9,009 |  | 3.5 | 590 |  | 0.7 | 187 |  | 1.5 | 8,899 |  | 3.3 | 706 |
| *Wealth quintile* |  |  |  |  |  |  |  |  |  |  |  |  |  |  |  |  |  |
| Poorest | 34.5 | 9,710 |  | 21.1 | 1,30,919 |  | 11.9 | 2,013 |  | 34.2 | 9,147 |  | 21.1 | 1,29,739 |  | 14.2 | 3,071 |
| Poorer | 23.7 | 6,652 |  | 20.3 | 1,26,015 |  | 11.3 | 1,900 |  | 23.7 | 6,329 |  | 20.4 | 1,25,177 |  | 12.5 | 2,699 |
| Middle | 19.1 | 5,369 |  | 20.2 | 1,25,443 |  | 12.9 | 2,165 |  | 19.1 | 5,103 |  | 20.3 | 1,24,663 |  | 13.6 | 2,934 |
| Richer | 13.1 | 3,684 |  | 19.6 | 1,21,713 |  | 19.3 | 3,247 |  | 13.2 | 3,527 |  | 19.7 | 1,20,865 |  | 19.1 | 4,113 |
| Richest | 9.7 | 2,715 |  | 18.7 | 1,15,742 |  | 44.7 | 7,526 |  | 9.8 | 2,605 |  | 18.6 | 1,14,675 |  | 40.6 | 8,748 |
| **Total** |  | **28,130** |  |  | **6,19,832** |  |  | **16,851** |  |  | **26,711** |  |  | **6,15,118** |  |  | **21,565** |
| **Note**: HH-Household | | | | | | | | | | | | | | | | | |

**Table S4**: Association between disease awareness status of members within clustered households and selected household characteristics: estimates from the multinomial regression

|  | **Hypertension** | | | | | | | | |  | **Diabetes** | | | | | | | | |
| --- | --- | --- | --- | --- | --- | --- | --- | --- | --- | --- | --- | --- | --- | --- | --- | --- | --- | --- | --- |
|  | **Mixed Awareness** | | | |  | **All unaware** | | | |  | **Mixed Awareness** | | | |  | **All unaware** | | | |
|  | **RRR** | **p-value** | **95% CI** | |  | **RRR** | **p-value** | **95% CI** | |  | **RRR** | **p-value** | **95% CI** | |  | **RRR** | **p-value** | **95% CI** | |
| *Number of 15-30 years members in the HH* | 1.19 | <0.001 | 1.11 | 1.28 |  | 1.33 | <0.001 | 1.23 | 1.44 |  | 1.11 | 0.139 | 0.97 | 1.27 |  | 1.24 | 0.001 | 1.09 | 1.41 |
| *Number of 31-50 years members in the HH* | 1.46 | <0.001 | 1.35 | 1.57 |  | 1.34 | <0.001 | 1.24 | 1.46 |  | 1.58 | <0.001 | 1.39 | 1.80 |  | 1.45 | <0.001 | 1.29 | 1.65 |
| *Number of 51-60 years members in the HH* | 1.39 | <0.001 | 1.24 | 1.55 |  | 1.27 | <0.001 | 1.13 | 1.44 |  | 1.29 | 0.008 | 1.07 | 1.57 |  | 1.25 | 0.020 | 1.03 | 1.50 |
| *Number of 61-70 years members in the HH* | 0.94 | 0.337 | 0.83 | 1.07 |  | 0.90 | 0.151 | 0.79 | 1.04 |  | 0.80 | 0.043 | 0.64 | 0.99 |  | 0.70 | 0.001 | 0.56 | 0.86 |
| *Share of 15+ years female members in the HH* | 0.99 | 0.001 | 0.98 | 0.99 |  | 0.99 | <0.001 | 0.98 | 0.99 |  | 0.99 | 0.702 | 0.99 | 1.01 |  | 0.99 | 0.358 | 0.98 | 1.01 |
| *Place of residence* |  |  |  |  |  |  |  |  |  |  |  |  |  |  |  |  |  |  |  |
| Urban ® |  |  |  |  |  |  |  |  |  |  |  |  |  |  |  |  |  |  |  |
| Rural | 1.04 | 0.607 | 0.89 | 1.22 |  | 1.28 | 0.006 | 1.07 | 1.52 |  | 1.17 | 0.227 | 0.90 | 1.53 |  | 1.39 | 0.012 | 1.07 | 1.79 |
| *Sex of the HH head* |  |  |  |  |  |  |  |  |  |  |  |  |  |  |  |  |  |  |  |
| Male ® |  |  |  |  |  |  |  |  |  |  |  |  |  |  |  |  |  |  |  |
| Female | 1.28 | 0.041 | 1.01 | 1.62 |  | 1.15 | 0.296 | 0.89 | 1.48 |  | 1.33 | 0.197 | 0.86 | 2.06 |  | 1.13 | 0.561 | 0.74 | 1.74 |
| *Schooling of HH head* |  |  |  |  |  |  |  |  |  |  |  |  |  |  |  |  |  |  |  |
| No schooling ® |  |  |  |  |  |  |  |  |  |  |  |  |  |  |  |  |  |  |  |
| Primary | 0.99 | 0.990 | 0.82 | 1.22 |  | 0.77 | 0.013 | 0.63 | 0.95 |  | 1.05 | 0.817 | 0.70 | 1.56 |  | 0.77 | 0.168 | 0.53 | 1.12 |
| Secondary | 0.93 | 0.441 | 0.77 | 1.12 |  | 0.77 | 0.009 | 0.64 | 0.94 |  | 1.12 | 0.545 | 0.78 | 1.61 |  | 0.75 | 0.100 | 0.54 | 1.06 |
| Higher | 0.88 | 0.303 | 0.69 | 1.12 |  | 0.71 | 0.012 | 0.54 | 0.93 |  | 0.94 | 0.787 | 0.61 | 1.45 |  | 0.64 | 0.037 | 0.43 | 0.98 |
| *Caste of HH head* |  |  |  |  |  |  |  |  |  |  |  |  |  |  |  |  |  |  |  |
| Scheduled Tribe ® |  |  |  |  |  |  |  |  |  |  |  |  |  |  |  |  |  |  |  |
| Scheduled Caste | 0.91 | 0.541 | 0.67 | 1.23 |  | 0.62 | 0.002 | 0.46 | 0.84 |  | 1.39 | 0.286 | 0.76 | 2.57 |  | 0.75 | 0.331 | 0.43 | 1.33 |
| Other Backward Classes | 0.72 | 0.016 | 0.55 | 0.94 |  | 0.44 | <0.001 | 0.33 | 0.57 |  | 0.94 | 0.817 | 0.56 | 1.58 |  | 0.41 | <0.001 | 0.26 | 0.66 |
| Others | 0.63 | 0.001 | 0.48 | 0.82 |  | 0.35 | <0.001 | 0.27 | 0.46 |  | 1.11 | 0.705 | 0.66 | 1.85 |  | 0.58 | 0.026 | 0.36 | 0.94 |
| *Religion* |  |  |  |  |  |  |  |  |  |  |  |  |  |  |  |  |  |  |  |
| Other religion ® |  |  |  |  |  |  |  |  |  |  |  |  |  |  |  |  |  |  |  |
| Hindu | 0.93 | 0.764 | 0.60 | 1.45 |  | 1.47 | 0.103 | 0.93 | 2.34 |  | 1.59 | 0.347 | 0.60 | 4.20 |  | 1.03 | 0.940 | 0.44 | 2.43 |
| Muslim | 0.65 | 0.088 | 0.40 | 1.07 |  | 1.07 | 0.814 | 0.63 | 1.80 |  | 1.49 | 0.459 | 0.52 | 4.24 |  | 0.91 | 0.839 | 0.36 | 2.31 |
| Christian | 0.63 | 0.050 | 0.39 | 1.00 |  | 0.68 | 0.127 | 0.41 | 1.12 |  | 1.27 | 0.648 | 0.46 | 3.52 |  | 0.46 | 0.100 | 0.19 | 1.16 |
| Sikh | 1.03 | 0.905 | 0.60 | 1.78 |  | 1.42 | 0.247 | 0.79 | 2.57 |  | 1.23 | 0.723 | 0.39 | 3.85 |  | 0.68 | 0.470 | 0.24 | 1.93 |
| *Wealth quintile* |  |  |  |  |  |  |  |  |  |  |  |  |  |  |  |  |  |  |  |
| Poorest ® |  |  |  |  |  |  |  |  |  |  |  |  |  |  |  |  |  |  |  |
| Poorer | 0.80 | 0.117 | 0.61 | 1.06 |  | 0.45 | <0.001 | 0.35 | 0.59 |  | 0.76 | 0.427 | 0.39 | 1.50 |  | 0.43 | 0.007 | 0.23 | 0.79 |
| Middle | 0.65 | 0.002 | 0.50 | 0.85 |  | 0.31 | <0.001 | 0.24 | 0.40 |  | 0.53 | 0.049 | 0.28 | 0.99 |  | 0.25 | <0.001 | 0.14 | 0.45 |
| Richer | 0.51 | <0.001 | 0.39 | 0.67 |  | 0.20 | <0.001 | 0.15 | 0.27 |  | 0.46 | 0.016 | 0.24 | 0.86 |  | 0.17 | <0.001 | 0.10 | 0.31 |
| Richest | 0.49 | <0.001 | 0.37 | 0.66 |  | 0.17 | <0.001 | 0.12 | 0.23 |  | 0.39 | 0.004 | 0.20 | 0.74 |  | 0.10 | <0.001 | 0.06 | 0.19 |
| Constant | 3.65 | <0.001 | 2.09 | 6.36 |  | 7.78 | <0.001 | 4.35 | 13.92 |  | 1.33 | 0.665 | 0.37 | 4.75 |  | 23.34 | <0.001 | 7.42 | 73.39 |
| **Note**: All aware is treated as the base outcome; RRR-Relative Risk Ratio; CI-Confidence Interval; HH-Household | | | | | | | | | | | | | | | | | | | |

**Checklist S1:** STROBE checklist of items that should be included in reports of **cross-sectional studies**

|  | Item No. | Recommendation | Section, paragraph Response | Page |
| --- | --- | --- | --- | --- |
| **Title and abstract** | 1 | (*a*) Indicate the study’s design with a commonly used term in the title or the abstract | Title | 1 |
|  |  | (*b*) Provide in the abstract an informative and balanced summary of what was done and what was found | Abstract | 2 |
| Introduction | | |  |  |
| Background/ rationale | 2 | Explain the scientific background and rationale for the investigation being reported | Abstract, Introduction | 2, 5 |
| Objectives | 3 | State specific objectives, including any prespecified hypotheses | Abstract, Introduction: final paragraph | 2, 7 |
| Methods | | |  |  |
| Study design | 4 | Present key elements of study design early in the paper | Abstract, Methods: Data Source, Measurement, Analytical procedure | 2, 7, 8, 9, 10 |
| Setting | 5 | Describe the setting, locations, and relevant dates, including periods of recruitment, exposure, follow-up, and data collection | Methods: Data Source, Measurement, Supplementary File 1 Text S1, Supplementary File 1 Text S2 | 7, 8 |
| Participants | 6 | (*a*) Give the eligibility criteria, and the sources and methods of selection of participants | Methods: Data Source, Measurement, Analytical procedure. | 7, 8, 9 |
| Variables | 7 | Clearly define all outcomes, exposures, predictors, potential confounders, and effect modifiers. Give diagnostic criteria, if applicable | Methods: Data Source, Measurement, Analytical procedure, Supplementary File 1 Text S3, Supplementary File 1 Text S4 | 7, 8, 9 |
| Data sources/ measurement | 8* | For each variable of interest, give sources of data and details of methods of assessment (measurement). Describe comparability of assessment methods if there is more than one group | Methods: Data Source, Measurement, Analytical procedure. Supplementary File 1 Text S1, Supplementary File 1 Text S2, Supplementary File 1 Text S3, Supplementary File 1 Text S4 | 7, 8, 9 |
| Bias | 9 | Describe any efforts to address potential sources of bias | Methods: Data Source, Measurement, Analytical procedure. | 7, 8, 9 |
| Study size | 10 | Explain how the study size was arrived at | Methods: Data Source, Measurement, Analytical procedure. Results: first paragraph. Supplementary File 1 Figure S1 | 7, 8, 9, 10, 11 |
| Quantitative variables | 11 | Explain how quantitative variables were handled in the analyses. If applicable, describe which groupings were chosen and why | Methods: Analytical procedure, Supplementary File 1Table S2 | 9, 10 |
| Statistical methods | 12 | (*a*) Describe all statistical methods, including those used to control for confounding | Methods: Analytical procedure | 8, 9, 10 |
|  |  | (*b*) Describe any methods used to examine subgroups and interactions | Methods: Analytical procedure | 8, 9, 10 |
|  |  | (*c*) Explain how missing data were addressed | Methods: Data Source, Supplementary File 1 Table S3 | 7 |
|  |  | (*d*) If applicable, describe analytical methods taking account of sampling strategy | Methods: Analytical procedure last paragraph | 10 |
|  |  | (*e*) Describe any sensitivity analyses | Not applicable |  |
| Results | | |  |  |
| Participants | 13 | (a) Report numbers of individuals at each stage of study—eg numbers potentially eligible, examined for eligibility, confirmed eligible, included in the study, completing follow-up, and analysed | Supplementary File 1 Figure S1, Results: First paragraph | 11 |
|  |  | (b) Give reasons for non-participation at each stage | Supplementary File 1 Figure S1, Results: First paragraph | 11 |
|  |  | (c) Consider use of a flow diagram | Supplementary File 1 Figure S1 |  |
| Descriptive data | 14* | (a) Give characteristics of study participants (eg demographic, clinical, social) and information on exposures and potential confounders | Supplementary File 1 Table S2 |  |
|  |  | (b) Indicate number of participants with missing data for each variable of interest | Not applicable |  |
| Outcome data | 15* | Report numbers of outcome events or summary measures | Table 1, Figure 1, Table 3, and Table 4 | 24, 26, 27, 28 |
| Main results | 16 | (*a*) Give unadjusted estimates and, if applicable, confounder-adjusted estimates and their precision (eg, 95% confidence interval). Make clear which confounders were adjusted for and why they were included | Table 1, Figure 1, Table 2, Table 3, and Table 4 | 24, 25, 26, 27, 28 |
|  |  | (*b*) Report category boundaries when continuous variables were categorized | Not applicable |  |
|  |  | (*c*) If relevant, consider translating estimates of relative risk into absolute risk for a meaningful time period | Not Applicable |  |
| Other analyses | 17 | Report other analyses done—eg analyses of subgroups and interactions, and sensitivity analyses | Not Applicable |  |
| Discussion | | |  |  |
| Key results | 18 | Summarise key results with reference to study objectives | Abstract, What this study adds, Discussion | 2, 3, 14 |
| Limitations | 19 | Discuss limitations of the study, taking into account sources of potential bias or imprecision. Discuss both direction and magnitude of any potential bias | Methods, Discussion: paragraph 7 | 7, 16 |
| Interpretation | 20 | Give a cautious overall interpretation of results considering objectives, limitations, multiplicity of analyses, results from similar studies, and other relevant evidence | Discussion | 14, 15, 16, 17 |
| Generalisability | 21 | Discuss the generalisability (external validity) of the study results | Discussion: final paragraph 8 | 17 |
| Other information | | |  |  |
| Funding | 22 | Give the source of funding and the role of the funders for the present study and, if applicable, for the original study on which the present article is based | Foot notes | 18 |
